# The Cognitive Profile of Gifted Children Compared to Those of Their Parents: A Descriptive Study Using the Wechsler Scales

**DOI:** 10.3390/jintelligence10040091

**Published:** 2022-10-24

**Authors:** Lina Pezzuti, Morena Farese, James Dawe, Marco Lauriola

**Affiliations:** 1 Department of Dynamic and Clinical Psychology and Health Studies, Sapienza University of Rome, 00185 Rome, Italy; 2 Department of Social and Developmental Psychology, Sapienza University of Rome, 00185 Rome, Italy

**Keywords:** giftedness, IQ, cognitive abilities, Wechsler scales, parent-children inheritance

## Abstract

The manifestation of performance at the top of a given talent distribution constitutes giftedness. While identifying talented youths based on IQ has been the focus of previous research, examining their cognitive profile is a new endeavor. The present study assessed the IQ and cognitive abilities of a sample of gifted Italian children and compared them to their parents using the Wechsler scales. Fifty-nine gifted children aged 6 to 14 years were administered the WISC-IV while their parents (*N* = 53 mothers and *N* = 55 fathers) took the WAIS-IV. The gifted children (IQ ≥ 120) obtained particularly high scores in verbal comprehension (VCI) and visual-perceptual reasoning (PRI). More than two-thirds of the mothers and over half of the fathers also achieved an IQ ≥ 120. The gifted children scored significantly higher than both mothers and fathers in VCI and PRI. The mothers were significantly higher than their children in the processing speed domain. Correlational analyses highlighted that children’s IQ was positively related to that of their mothers. In keeping with the literature, the cognitive profile of gifted children was found to vary across cognitive abilities. It follows that the General Ability Index was the WISC-IV index that best matched the potential of gifted youths. Consistent with previous research, our study suggests that intellectual abilities, especially working memory and processing speed, are maintained and presumably passed on from one generation to the next.

## 1. Introduction

There is general agreement that giftedness is a complex pattern of genetic, personal, and behavioral characteristics that result in exceptional abilities manifested in different ways in one or more areas considered to be prominent at a given point in time in one’s culture of belonging (e.g., Keating 2009; Pfeiffer 2012; Worrell and Erwin 2011). General intellectual ability, specific scholastic aptitudes, creative thinking, leadership, and the visual and performing arts are the major areas of giftedness expression in the Western world.

### 1.1. The Evaluation of Giftedness in Children

Until recently, a high Intelligence Quotient (IQ) was the only trait thought to distinguish gifted individuals in the population. For example, Carman (2013) found that using a general ability test (often the only measure used) was the most popular way to identify gifted individuals in 62% of the studies. However, at present, a multi-component view of giftedness emerged. In addition to high IQ, gifted individuals were found to share other characteristics, although in varying modalities and intensity, such as having a wide range of interests, intense curiosity and thirst for knowledge, strong communication skills, intuition, problem-solving skills, advanced logical skills, imagination and creativity, deep sensitivity, and empathy (e.g., Zanetti 2017).

Although IQ represents only a partial expression of giftedness, according to a purely psychometric view, giftedness is defined by an IQ of 130 or higher, placing gifted individuals at least two standard deviations above the population mean. However, no shortage of authors extended this range, considering people who have an IQ below 130 as gifted individuals. For example, Ruf (2005) proposed five levels of giftedness, with individuals with IQ scores between 117 and 129 being considered “moderately gifted”. Not too dissimilar to Ruf (2005), Silverman (2009; 2018) also proposed five levels of giftedness (i.e., mild, moderate, high, exceptional, and profound) starting at a minimum level of 120 IQ points. Both classifications are based on performance in psychometric tests. However, there is another reason to lower the IQ cutoff for giftedness below 130. For example, the most widely used studies on gifted education identify gifted scores in the range of 115–129 IQ points for students who are non-native English speakers or come from low-education families (e.g., Crabtree et al. 2019). In countries where linguistic and cultural minorities are less prevalent or more supported within a public education system, the use of IQ may be an all-too-fair criterion in screening for and assessing giftedness. However, it appears that the idea of maintaining a lower threshold than 130 IQ points or differentiating even wider performance bands is gaining traction. For example, the definition of “high-potential” individuals with IQ scores between 120 and 129 could be used (Sartori 2019; Zanetti 2017) precisely because during the assessment they showed the potential to excel while obtaining an IQ score below 130 on standardized tests.

One of the best-known and most widely used instruments for assessing the cognitive abilities of children and adolescents is the Wechsler Intelligence Scale for Children-Fourth Edition (WISC-IV). Analysis of the WISC-IV cognitive profiles of gifted children revealed that these children’s greatest cognitive strengths were in verbal reasoning, visual perception, and fluid reasoning (e.g., Liratni and Pry 2007, 2012; Rimm et al. 2008; Silverman 2009). In contrast, working memory ability and speed of cognitive processing, despite being typically higher than the general population average, were found to be a “weak” point in the profile of the gifted (e.g., Morrone et al. 2019; Rimm et al. 2008; Rowe et al. 2014). These findings have suggested that gifted children’s preference for accuracy over speed may account for their relatively lower performance in cognitive processing speed tests (Morrone et al. 2019). Indeed, gifted children are thought to work meticulously, implementing frequent self-monitoring mechanisms that can penalize execution speed.

Discrepancies in the cognitive profiles of the gifted have also led scholars to speculate that the full-scale IQ is by no means the cognitive index that best captures the complexity of their intellectual endowment (Morrone et al. 2019; Rimm et al. 2008; Rowe et al. 2014; Silverman 2009; Sparrow et al. 2005). In place of or in addition to the full-scale IQ, it has been suggested that the WISC-IV General Ability Index be considered to minimize the impact of speed and memory subtests in which gifted children tend to perform below their average (Hagmann-von Arx et al. 2008; Rimm et al. 2008; Silverman 2009; Sparrow et al. 2005). For example, in an Italian study (Toffalini et al. 2017) involving a large sample of gifted children with a specific learning disorder, giftedness emerged more clearly when the General Ability Index was used.

However, not all researchers agree. For example, Rowe et al. (2014) maintain that neither the IQ nor the General Ability Index can capture the complexity of the intellectual profile of the gifted. As a result, these authors advocated for the use of the WISC-IV broad abilities, which can explain more than 60% of the variance in the gifted cognitive profile.

### 1.2. Children’s Giftedness and Parents’ Cognitive Abilities

While studies that have focused on intellectual endowment have multiplied over the past few decades, there is still very limited knowledge about the cognitive characteristics of the parents of gifted children. Since earlier anecdotal observations made by Galton, the implementation and development over the past century of increasingly better-designed studies with larger and more generalizable samples have allowed researchers to conclude that about half of the variability in human intelligence can be explained by hereditary factors transmitted from one generation to the next (Deary et al. 2006, 2009; Guez et al. 2021). Furthermore, while it would seem more reasonable to expect that the influence of heredity would diminish as new experiences accumulate over the course of one’s life, genetic studies have revealed that gene-related variability in intelligence test scores rises from around 30% in childhood to 80% in adulthood (Deary et al. 2009).

Geneticists believe that the high heritability of intelligence is due to a phenomenon known as assortative mating, which can be explained as follows: two partners with similar cognitive abilities are more likely to choose each other than by chance. Data reported by several studies (Deary et al. 2006; Guez et al. 2021; Plomin and Deary 2015; Plomin and Spinath 2004) are consistent with this view. For example, when the personality characteristics of the spouses are analyzed, the correlations between partners are null or small (r ≈ .10); in contrast, when their cognitive abilities are examined, the correlations are moderate (up to r ≈ .40) (Deary et al. 2006; Plomin and Deary 2015; Plomin and Spinath 2004).

If about half of the variability in human intelligence can be explained by hereditary factors (Deary et al. 2006, 2009), the remaining part can be due to environmental factors. Thus, there has been increasing evidence supporting the importance of shared environmental influence in the development of intellectual abilities (Guez et al. 2021). For example, when comparing parents and adopted children, the correlations were found to be small (r ≈ .19), while comparing parents and biological children, the correlation was higher (r ≈ .41) (Plomin and Spinath 2004). Because adoptive siblings have different genetics, what undoubtedly made them similar was sharing the same family environment. However, the many efforts made to identify the specific environmental characteristics that can influence cognitive development have been unsuccessful, although the contribution of some socioeconomic variables has not been ruled out (Plomin and Spinath 2004).

When researchers examined the concordance of parents’ cognitive level with that of their children, the data collected suggested that there is greater consistency in mother-child dyads than in father-child dyads (Anger and Heineck 2010; Calderon and Hoddinott 2010; Demange et al. 2022; Grönqvist et al. 2010). For example, Anger and Heineck (2010) showed that the correlations of IQ between parents and children were r ≈ .34 for father-daughter dyads and r ≈ .48 for mother-daughter dyads. Similarly, Grönqvist et al. (2010) assessed father-son correlations to be r ≈ .51, while mother-son correlations were found to be r ≈ .59. The same study also assessed the parent-child similarities in non-cognitive variables, concluding that mother-child and father-child correlations were more similar (i.e., r ≈ .46).

Recently, researchers coined the term “gene-environment interaction” to combine genetic data with those that support the influence of the environment on cognitive development (Sauce and Matzel 2018). This approach, which led to the abolition of the now outdated dichotomy between nature and culture, had an even greater benefit: it restored the vision of humanity not destined to be influenced by genes or social background but capable, if necessary, of selecting, modifying, or creating from scratch a living environment in which to express its potential (Plomin and Spinath 2004; Sauce and Matzel 2018).

### 1.3. The Present Study

The main objective of the present study is to investigate the similarities and differences in the cognitive profiles of gifted children and their parents in general intellectual ability and broad cognitive abilities. In particular, the present study takes advantage of the Wechsler scales (WISC-IV and WAIS-IV), which have been not only widely used to assess giftedness in children and adults but also have similar structural characteristics and psychometric properties. Indeed, using the Wechsler scale, one can obtain an IQ, four primary indices relative to broad abilities (verbal comprehension, perceptual reasoning, working memory, and processing speed), and two supplementary indices reflecting the general ability and the cognitive proficiency of the profile.

First, we assessed the cognitive profile of a sample of children, with an IQ ≥ 120 on the WISC-IV, hypothesizing that their cognitive abilities would be relatively higher in the domains of verbal comprehension and perceptual reasoning while obtaining relatively lower scores in working memory and processing speed (Morrone et al. 2019; Rimm et al. 2008; Rowe et al. 2014; Silverman 2009). Consequently, we expected the children’s profile to be characterized by relatively higher general ability than cognitive proficiency (Toffalini et al. 2017).

In a similar vein, we assessed the cognitive profiles of the parents using the WAIS-IV. In keeping with previous research using typically developing samples (Deary et al. 2009; Plomin and Spinath 2004; Plomin and von Stumm 2018), we hypothesized that a large percentage of parents of gifted children would obtain a higher IQ than the general population as well as primary and supplementary indices scores (especially in verbal comprehension, perceptual reasoning, and general ability) above the average of the national standardization sample. To the best of our knowledge, no previous study surveyed a sample of parents of gifted children and compared them in the same broad ability areas. Therefore, the data we presented in this paper represent an element of novelty and have no terms of comparison in the literature.

Finally, we compared the profile of parents to that of their gifted children. Based on the reviewed literature (Deary et al. 2006, 2009; Plomin and Spinath 2004; Plomin and von Stumm 2018), we expected a positive, moderate correlation between the corresponding IQ scores of the WISC-IV and the WAIS-IV. Previous research has not only pointed out that intellectual abilities are passed on from one generation to the next (Bouchard and McGue 1981; Deary et al. 2006) but also that there is a greater concordance between the cognitive level of mothers and children than between the cognitive level of fathers and children (Anger and Heineck 2010; Grönqvist et al. 2010). If any, we expect greater correlations in mother-child dyads than in father-child ones.

To the best of our knowledge, no previous study has compared gifted children to their parents in broad cognitive abilities, such as the primary and additional indices of the Wechsler scales. Therefore, another novel aspect of this study is to analyze and describe the performance trends in the subdomains of intelligence in parents and gifted children.

## 2. Materials and Methods

### 2.1. Participants

Participants were recruited through advertisements on the websites of parents’ associations of gifted children and related social network groups. Upon learning about the study, all participating families chose to join voluntarily and without compensation. To confirm that eligible children qualified as moderately gifted or gifted, they had to score between 120 and 129 for the former and 130 or higher for the latter on the FSIQ of the WISC-IV. As a result, 59 MG or gifted children and adolescents (19 girls and 40 boys) aged between 6 and 14 years (M = 10.03 years; SD = 2.18 years) were identified and recruited for the present study. Boys (M = 10.40 years; SD = 2.02 years) were older than girls (M = 9.26 years; SD = 2.35 years), but this difference was not statistically significant (t = −1.91, df = 57, *p* = .060). Moreover, no gender differences were found in the children’s IQ. All children attended public schools in Italy, and the majority of the sample was comprised of middle-class families.

The parents of each child were also tested using the WAIS-IV, and data were collected from 53 mothers and 55 fathers. Mother’s age ranged from 36 to 55 years (M = 43.70 years; SD = 4.11 years), while father’s age ranged from 36 to 70 years (M = 45.75 years; SD = 5.48 years), t(48) = −3.51, *p* < .001, Cohen’s d = −.50. Mothers were overall more educated than fathers (t = 6.45, df = 54, *p* < .001; Cohen’s d = .87), see Table 1 for parents’ educational qualifications. The mothers’ IQ scores were also higher than that obtained by the fathers (t = 2.35, df = 48, *p* = .023; Cohen’s d = .34). Wechsler scales were administered specifically for this study, and data were not obtained via prior records. There were no administration differences due to setting (e.g., private testing vs. school-based administration). Both children and parents were evaluated under standardized conditions at the psychological testing laboratory of the Department of Dynamic, Clinical and Health Studies of Sapienza University of Rome. Before testing, all participants provided written informed consent and were recruited voluntarily. The study was approved by the local ethical committee at the Department of Dynamic, Clinical and Health Studies of Sapienza University of Rome. 

### 2.2. Instruments

Wechsler Intelligence Scale for Children-Fourth Edition-Italian version (WISC-IV; Orsini et al. 2012). The WISC-IV is an instrument aimed at assessing the cognitive abilities of children and adolescents between the ages of 6 and 16. The scale, through the administration of 15 subtests-10 basic and 5 supplementary-provides a Full-Scale IQ (FSIQ), putatively reflecting the g-factor of intelligence, and four primary indices corresponding to broad cognitive abilities (i.e., Verbal Comprehension Index, or VCI; Perceptual Reasoning Index, or PRI; Working Memory Index, or WMI; and Processing Speed Index, or PSI). Furthermore, two supplementary indices can be derived: the General Ability Index (GAI), which is an estimate of intellectual functioning obtained by six subtests of VCI and PRI only, and the Cognitive Proficiency Index (CPI), which reflects efficient information processing obtained by four subtests of WMI and PSI (e.g., Kaufman et al. 2006; Rimm et al. 2008; Watkins et al. 2006).

Wechsler Adult Intelligence Scale-Fourth Edition-Italian version (WAIS-IV; Orsini and Pezzuti 2013). The WAIS-IV is a clinical tool used to assess the cognitive abilities of people ranging in age from 16 to 90 years. It consists of the following 15 subtests (10 core and five supplementary): Similarities, Vocabulary, Information, Comprehension, Block Design, Matrix Reasoning, Visual Puzzles, Figure Weights, Figure Completion, Digit Span, Arithmetic, Letter-Number Sequencing, Symbol Search, Coding, and Cancellation. Like the previously described WISC-IV, the WAIS-IV yields four primary indices, each of which corresponds to a broad ability area (i.e., VCI, PRI, WMI, and PSI). Averaging the four indices, one can obtain the FSIQ, while GAI and CPI can be obtained by subtests of VCI and PRI, and WMI and PSI, respectively.

### 2.3. Data Analysis

The frequency distributions of children’s and parents’ Wechsler scale scores were examined for violations of normality. The Shapiro–Wilk’s test was significant for the children’s FSIQ (*p* = .018), the mother’s GAI (*p* = .017), and the father’s FSIQ (*p* = .030), VCI (*p* = .004), and GAI (*p* = .002). However, the distributions were only moderately asymmetrical, with a skewness between −1.0 and 1.0 for all variables. The Kurtosis was between −3 and 3 for all variables, indicating that extreme values were not very different from those expected according to normal data distribution. Thus, although some significant deviations from the assumption of normality were observed, none of the variables examined was of concern.

To examine the cognitive profile of the research participants, descriptive analyses were conducted both on continuous IQ scores and by score categories. Because there is no consensus in the literature on the width of intervals for classifying giftedness, sometimes resorting to arbitrary intervals of varying widths depending on IQ level (e.g., Wasserman 2013), we used 10-point intervals for the analysis in this study. This decision was made based on the descriptive classification proposed in the Italian WISC-IV standardization manual (Orsini et al. 2012), which is referred to in the American WISC-IV technical and interpretive manual (Wechsler 2003). When using the Wechsler scales, we believe that these intervals have greater sensitivity than the 15-point intervals for highlighting variability in children’s cognitive abilities. Therefore, the following four levels of performance were considered for each of the Wechsler scale scores: (1) *Gifted*, if the score was greater than or equal to 130; (2) *Moderately Gifted*, if the score was between 120 and 129; (3) *Above average*, if the score was between 110 and 119; (4) *Average*, if the score was below 110.

A multivariate analysis of variance (MANOVA) was conducted on the Wechsler scores in the Children sample, while in the Mothers and Fathers sample, the same analysis included parental gender as a between-subjects factor. These analyses were aimed to investigate whether and which ability was relatively higher than others in the cognitive profile of each participant. Bonferroni-corrected post hoc tests were calculated to evaluate within-subjects differences and to compare mothers to fathers on specific IQ scores.

To examine the associations between children’s IQ scores and both mother’s and father’s scores, a series of paired *t*-tests analyzed dyadic differences, contrasting mother and children, and father and children, respectively. Pearson and intraclass correlations were used to establish the degree of similarity between children and parent dyads.

## 3. Results

### 3.1. The Cognitive Profile of Gifted Children

The frequencies with which the seven WISC-IV indices fell in the average, above average, moderately gifted, and gifted performance ranges were calculated for the entire sample and reported in Table 2. According to the selection criteria, all participants had an FSIQ ≥ 120. This notwithstanding, the analyses of the WISC-IV primary indices showed that most participants were just in the average or above average ranges in the working memory and processing speed indices (WMI and PSI, respectively). Only the distribution of verbal comprehension and perceptual reasoning indices (VCI and PRI) mirrored quite well that of the FSIQ. As a result, the distribution of scores based on the two supplemental indices showed that all children had a General Ability Index (GAI) ≥ 120, while less than 40% had a Cognitive Proficiency Index (CPI) in the moderately gifted or gifted ranges.

**Figure 1:**
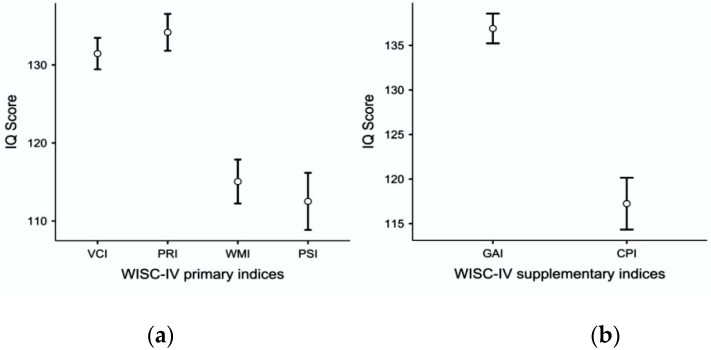
The cognitive profile of Moderately Gifted and Gifted children using (**a**) WISC-IV primary indices; (**b**) WISC-IV supplementary indices. Whiskers represent the 95% confidence interval (CI), and circles represent the average score obtained by the sample.

Figure 1 shows the cognitive profile of the children using the WISC-IV primary and supplementary indices. The within-subject variability of primary indices was statistically significant (F_3,174_ = 63.00; *p* < .001; partial η^2^ = .52). Bonferroni-corrected post hoc tests (reported in Table 3) showed that verbal comprehension and perceptual reasoning abilities were superior to working memory ability and speed of cognitive processes, while verbal comprehension and perceptual reasoning did not differ from each other (as did working memory and speed of cognitive processes). Again, these findings suggested that Moderately Gifted and Gifted children performed better on tasks that require an understanding of verbal information, thinking and reasoning with words, expressing thoughts as words, solving nonverbal problems, using eye-hand coordination, and working quickly and efficiently with visual information (Hagmann-von Arx et al. 2008; Morrone et al. 2019; Rimm et al. 2008; Rowe et al. 2014). Consequently, the GAI outperformed the CPI in the children sample (F_3,174_ = 109.01; *p* < .001; partial η^2^ = .65). Indeed, the GAI was greater than 130 (i.e., two standard deviations above the average IQ), while the CPI was just around 115 (i.e., one standard deviation above the average IQ).

Next, we counted the number of WISC-IV indices in which each child scored ≥ 120. As shown in Table 4, all children had at least one index in the gifted range. However, most of the sample (47.5%) reached or exceeded a score of 120 only in two out of four indices. Only nine children (15.3%) scored higher than or equal to 120 in all four broad ability areas of the WISC-IV. In addition, while 81% of the subjects achieved an IQ score ≥ 120 in both CVI and PRI, only 15% achieved the same result in both WMI and PSI. These results represent further evidence that giftedness does not necessarily involve excellent performance in all ability areas, at least those examined by the WISC-IV scale.

### 3.2. The Cognitive Profile of Parents of Moderately Gifted or Gifted Children

Replicating Table 2, Table 5 reports the frequencies with which the seven WAIS-IV indices fell in the average, above average, moderately gifted, and gifted performance ranges for the maternal and paternal samples. Approximately 68% of the mothers and 58% of the fathers had an FSIQ ≥ 120. With the only exception of WMI, the percentage of mothers scoring ≥ 120 in the remaining WAIS-IV indices of the scale was higher than the corresponding percentage of fathers.

**Figure 2:**
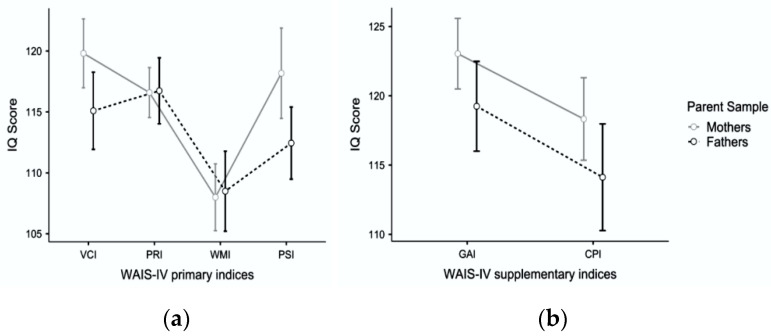
The cognitive profile of mothers and fathers of Moderately Gifted and Gifted children using (**a**) WAIS-IV primary indices; (**b**) WAIS-IV supplementary indices. Whiskers represent the 95% confidence interval (CI), and circles represent the average score obtained by each sample.

Figure 2 shows the cognitive profile of the mothers and fathers of moderately gifted and gifted children using the WAIS-IV primary and supplementary indices. The analysis of primary indices (Figure 2a) showed that the cognitive profile of mothers was overall higher than that of fathers (F_1,48_ = 5.17; *p* = .028; partial η^2^ = .10), and the within-subject variability of primary indices was also statistically significant (F_3,144_ = 21.78; *p* <.001; partial η^2^ = .52). In contrast, the sample × index interaction did not reach the conventional levels of significance (F_3,144_ = 2.19; *p* = .092; partial η^2^ = .04). Both in the mothers’ sample and the fathers’ sample, the verbal comprehension ability and the perceptual reasoning performance were superior to working memory ability.

Unlike their children’s cognitive profiles, the speed of cognitive processes in both parent samples was on par with verbal comprehension and perceptual reasoning (Table 6, panels a and b). In the mothers’ sample only, the speed of cognitive processes was also significantly greater than working memory (Table 6a).

The analysis of supplementary indices (Figure 2b) confirmed the overall higher elevation of the Mother’s profile (F_1,48_ = 5.14; *p* = .028; partial η^2^ = .10) as well as the significance of the within-subject factor, with both superior performances obtained in the GAI than in the CPI (F_1,48_ = 27.29; *p* < .001; partial η^2^ = .36). The sample × index interaction was not significant (F_1,48_ = 0.03; *p* = .865; partial η^2^ = .00). Consequently, the GAI outperformed the CPI in the parent samples as well as in the children sample.

### 3.3. Similarities and Differences in the Cognitive Profile of Parents and Children

A correlational analysis was carried out based on 53 mother-child and 55 father-child dyads to investigate the similarities between the parents’ cognitive profile and that of their moderately gifted and gifted children. As shown in Table 7, the correlations obtained from mother-child dyads were either statistically significant or approaching statistical significance. The intraclass correlations, representing the percentage of variance in the data accounted for by the dyad, ranged from 10% for VCI and GAI to 25% for PSI. The similarity in FSIQ between mothers and their children was estimated at 17%. Conversely, the correlations obtained from father-child dyads were near zero, except for PRI and WMI, approaching statistical significance only for the latter index. Because the mother’s level of education, but not that of the father, was found to influence the children’s intellectual performance (e.g., Kong et al. 2015), we also analyzed the partial correlations obtained from mother-child dyads controlling for the mother’s years of education. The partial correlations controlling for mother education were somewhat lower in size than zero-order correlations and remained statistically significant only for WMI (*r* = .23, *p* <.05) and PSI (*r* = .30, *p* <.05). Controlling also for father education, none of the partial correlations remained statistically significant, albeit approaching the conventional levels.

Subsequent analyses compared mother-child dyads to highlight discrepancies in the cognitive profile. As shown in Table 8, the largest differences were found in the VCI, PRI, GAI, and FSIQ, where the children outperformed their mothers by about 1SD in IQ points. In the PSI, mothers obtain significantly higher average scores than their children. Conversely, the cognitive profile of mothers and children was less discrepant in WMI and CPI.

As we compared father-child dyads (Table 9), we found even larger discrepancies in VCI, PRI, GAI, and FSIQ. Like the analysis of mother-child dyads, WMI, PSI, and CPI were noteworthy comparable in father-child dyads, where no statistical differences were found.

## 4. Discussion

The first objective of the present study was to examine the cognitive profile of gifted children, looking for discrepancies between the various skill areas. All children had at least one primary WISC-IV index in the moderately gifted or gifted range. However, only 15.3% of these children scored equal to or above 120 in all four broad abilities areas. Our study has shown that gifted children have a cognitive advantage in verbal comprehension and visual-perceptual domains. This result supports our hypotheses and adds to the existing knowledge about the intellectual talent of children and adolescents (Kaufman et al. 2006; Liratni and Pry 2007, 2012; Morrone et al. 2019; Rimm et al. 2008; Rowe et al. 2014; Silverman 2009). It is worth noting, however, that this general conclusion does not apply to all children in our sample. Some of them were found to obtain excellent working memory and processing speed performance. Future research should investigate the heterogeneity of the cognitive profile to verify the existence of different subpopulations of gifted children.

The literature also pointed out that processing speed is the relative-worst performance in the profile of gifted children, whereas the working memory ability, while not reaching the excellence of verbal and perceptual reasoning skills, is still far above average (e.g., Morrone et al. 2019). Working memory and processing speed did not show statistically significant differences in our study. Thus, our study is only partly consistent with Toffalini et al. (2017). However, in that study, gifted children were discovered among patients with a specific learning disorder whose working memory and processing speed were impaired, but not GAI, which was still excellent. Thus, a possible explanation for the discrepancy in the cognitive profile of the gifted children enrolled in our study and in previous research could be the different presence of “twice exceptional” children: i.e., gifted children with a specific learning disorder (Toffalini et al. 2017). Indeed, twice-exceptional children may show fragility in executive functions and working memory (Toffalini et al. 2017). Unfortunately, our study did not assess whether the eligible children (and their parents) received a learning disability disorder diagnosis. Thus, future research should consider a specific impairment in learning abilities as a condition that might account for the heterogeneity of the cognitive profile of the gifted.

Consistent with previous research (Hagmann-von Arx et al. 2008; Rimm et al. 2008; Silverman 2009; Sparrow et al. 2005), our study found that the GAI was higher than the FSIQ. Thus, while IQ could be useful as a psychometric standard to screen for gifted children, our study suggests that the GAI is more capable of highlighting the multiple skills and resources possessed by gifted individuals during an assessment, even when there may be dual or multiple exceptionalities.

The second goal of our research was to examine the cognitive profiles of parents of gifted children. First, it is worth noting that although all the children in our sample were selected based on an FSIQ ≥ 120, only 68% of mothers and 58% of fathers achieved the same criterion. Again, it is worth mentioning that using the GAI instead of the FSIQ, the similarities between the cognitive level of parents and their children increased, and even the average of the maternal sample far exceeded the 120 IQ point threshold, while that of the fathers’ sample came very close to the giftedness threshold. In the absence of comparative data in the literature, our results seem overall consistent with the results of genetic studies concluding that about half of the variability in human intelligence can be explained by hereditary factors (e.g., Deary et al. 2006, 2009; Plomin and Deary 2015).

Analysis of broad abilities using the primary and supplementary indices of the Wechsler scales showed that the cognitive profile of mothers was overall more elevated than that of fathers. In the mothers’ sample, the processing speed was significantly greater than working memory, placing itself on the same level as verbal comprehension and perceptual reasoning abilities. In the fathers’ sample, working memory was also a weakness in the cognitive profile. However, unlike mothers, the processing speed was not significantly different from working memory, although the latter was below verbal comprehension and perceptual reasoning abilities.

Although not all parents reached the giftedness threshold, their cognitive profile was similar to the intellectual profiles of gifted adults and elderly people, whose strength was in their high processing speed (Lang et al. 2019; Pezzuti et al. 2022). On the other hand, the cognitive profile of parents, and especially of mothers, was very dissimilar to that of their children precisely in relation to processing speed. Like their children, the parents also showed relatively lower performance in working memory ability. Just as we have discussed the drop in working memory in gifted children in relation to the literature on twice-exceptionality (Toffalini et al. 2017), similarly, we might hypothesize that a learning disorder related to executive functions may also be present but undiagnosed among parents.

The last goal of the present study was to identify similarities and differences in the cognitive profiles of parents and gifted children. The correlations obtained from the mother-child dyads were statistically significant or close to statistical significance and were higher than for the father-child dyads. In general, the correlations estimated in our study were somewhat lower than those typically reported in previous research with nongifted community samples (Anger and Heineck 2010; Calderon and Hoddinott 2010; Demange et al. 2022; Grönqvist et al. 2010). One factor that may have limited the magnitude of the correlations in our study might be traced to the rank restriction of total IQ, which we used as an entry criterion in the study. In contrast, the above research was conducted on large and representative samples of the population, with greater variability in general intelligence or in specific cognitive abilities. This notwithstanding, the similarity in FSIQ between mothers and children was estimated at 17%, while the correlations obtained from father-child dyads were near zero. These results appear to be consistent with data reported in the literature that compared the cognitive abilities of mothers and fathers with those of their typically developing children. More specifically, it was found that mothers’ cognitive abilities (e.g., executive functioning, working memory, verbal IQ) correlated with children’s cognitive abilities, whereas fathers’ non-cognitive abilities (e.g., academic motivation, perseverance, mindsets, learning strategies, and social skills) correlated with their children’s cognitive abilities (e.g., Demange et al. 2022; Grönqvist et al. 2010).

Inevitably, the current study has some limitations that, while necessitating caution in interpreting the findings, may aid in the development of new proposals for future research. First, we lack a control group of children with average IQs to compare to families with gifted children. This prevented us from ruling out other characteristics that might differentiate Italian-gifted children from their normal peers beyond IQ. Furthermore, it does not allow us to understand whether the profile of parents of gifted children is similar or different from that of parents of children with typical development, controlling for possible confounding factors such as the socioeconomic status of the families, the presence of possible cognitive impairment, and so forth. A follow-up to this research could be the recruitment of a control group of gifted families as similar as possible to those already recruited for the present research. Second, our study focused exclusively on the FSIQ of the WISC-IV, while in the gifted education literature, there are other criteria, such as qualitative judgments of giftedness or curriculum-based measures (e.g., Cao et al. 2017). Different results might emerge by identifying giftedness using different or alternative criteria than traditional psychometric methods. Other limitations are related to sampling and self-selection. Although all the children attended Italian public schools (in which no special curricula are directed to gifted children) and most of the families were of the middle class, we did not assess other specific environmental characteristics that—beyond parents’ education—can influence cognitive development (e.g., family income, family size, city neighborhood, etc.). For this reason, any bias due to socioeconomic variables cannot be ruled out. In addition, such analysis would be based on a very small total number of families and including parents who voluntarily participated in the present study. It is hoped that in the future, an attempt will be made to replicate this type of research with a larger and more widely distributed control and experimental sample. This could allow the identification of key variables that can help in defining different subgroups of gifted children with different cognitive profiles.

## 5. Conclusions

The identification of significant strengths (related to the verbal and visual-perceptual domains), as well as potential individual weaknesses in gifted children, can play a critical role in the proper implementation and planning of educational and support interventions. It is clear, in fact, how these children’s high levels of verbal comprehension and visual-perceptual skills may lead them to prefer specific learning methods in which new information is provided primarily through visual aids—such as tables, diagrams, and charts—accompanied by possible explanations and comments. Such approaches could allow moderately gifted and gifted learners to cope better with the proposed tasks and to maintain adequate levels of involvement in the required activities.

In terms of parental characteristics, the practical implications of this study are primarily concerned with the possibility of contributing to a better understanding of the relationship between parents and gifted children, as well as the possible implementation of specific parenting support paths. The high proportion of moderately gifted or gifted individuals among the parents explains why these families are frequently able to provide their children with a stimulating environment that nurtures their desire for knowledge, provides meaningful opportunities for growth, and respects their passions and interests.

Finally, one of the main strengths of this paper is that it has attempted to draw attention to certain cognitive aspects of parents that, if properly assessed and recognized, can instead become valuable allies in supporting all professionals involved in parenting support.

## Figures and Tables

**Table 1:** Frequency distribution of Educational qualification in mothers’ and fathers’ samples.

	*Mothers*		*Fathers*	
*Educational Qualification*	*n*	*%*	*n*	*%*
Junior high school	-	-	5	9.1
High school	10	18.8	24	43.6
Degree	25	47.2	23	41.8
Post-graduate	18	34.0	3	5.5
PhD	4	7.5	1	1.8
Master	9	17.0	1	1.8
Master + PhD	4	7.5	-	-
Other qualifications	1	19.0	1	1.8%
Total	53	100.0	55	100.0

**Table 2:** Frequency distribution of WISC-IV indices by children’s level of performance.

	Level of Performance							
Indices	Average (<110)		Above Average (110–119)		Moderately Gifted (120–29)		Gifted (≥130)	
	*n*	%	*n*	%	*n*	%	*n*	%
FSIQ	-	-	-	-	23	39.0	36	61.0
VCI	-	-	6	10.2	20	33.9	33	55.9
PRI	-	-	6	10.2	17	28.8	36	61.0
WMI	21	35.6	19	32.2	11	18.6	8	13.5
PSI	28	47.5	12	20.3	13	22.0	6	10.2
GAI	-	-	1	1.7	13	22.0	45	76.3
CPI	16	27.1	21	35.6	11	18.6	11	18.6

Legend: FSIQ = Full-Scale IQ; VCI = Verbal Comprehension Index; PRI = Perceptual Reasoning Index; WMI = Working Memory Index; PSI = Processing Speed Index; GAI = General Ability Index; CPI = Cognitive Proficiency Index (CPI). Note: *N* = 59.

**Table 3:** Bonferroni-corrected post hoc tests comparing the WISC-IV primary indices.

Comparison			Mean Difference	SE	df	t	p_bonferroni_
VCI	-	PRI	−2.73	1.74	58	−1.57	.737
VCI	-	WMI	16.41	1.94	58	8.48	<.001
VCI	-	PSI	18.95	2.35	58	8.06	<.001
PRI	-	WMI	19.14	1.61	58	11.86	<.001
PRI	-	PSI	21.68	2.14	58	10.11	<.001
WMI	-	PSI	2.54	1.99	58	1.28	1

Legend: FSIQ = Full-Scale IQ; VCI = Verbal Comprehension Index; PRI = Perceptual Reasoning Index; WMI = Working Memory Index; PSI = Processing Speed Index; GAI = General Ability Index; CPI = Cognitive Proficiency Index (CPI). Note: *N* = 59.

**Table 4:** Count of WISC-IV primary indices with a score ≥ 120.

Number of WISC-IV Primary Indices (≥120)	*n*	%
4	9	15.3
3	15	25.4
2	28	47.5
1	7	11.9
0	-	-
Total	59	100

**Table 5:** Frequency distribution of WAIS-IV indices by (a) mother’s and (b) father’s level of performance.

	**(a) Mother’s Level of Performance ^a^**							
**Indices**	**Average** **(<110)**		**Above Average** **(110–119)**		**Moderately Gifted** **(120–129)**		**Gifted** **(≥130)**	
	***n***	**%**	***n***	**%**	***n***	**%**	***n***	**%**
FSIQ	3	5.7	14	26.4	23	43.4	13	24.5
VCI	4	7.5	18	34.0	19	35.8	12	22.6
PRI	9	17.0	16	30.2	20	37.7	8	15.1
WMI	28	52.8	16	30.2	7	13.2	2	3.8
PSI	14	26.4	7	13.2	14	26.4	18	34.0
GAI	4	7.5	14	26.4	22	41.5	13	24.5
CPI	12	22.6	14	26.4	23	43.4	4	7.5
	**(b) Father’s Level of Performance ^b^**							
**Indices**	**Average** **(<110)**		**Above Average** **(110–119)**		**Moderately Gifted** **(120–129)**		**Gifted** **(≥130)**	
	***n***	**%**	***n***	**%**	***n***	**%**	***n***	**%**
FSIQ	14	25.5	9	16.4	16	29.1	16	29.1
VCI	15	27.3	10	18.2	26	47.3	4	7.3
PRI	7	12.7	26	47.3	15	27.3	7	12.7
WMI	31	56.4	9	16.4	12	21.8	3	5.5
PSI	20	36.4	17	30.9	9	16.4	9	16.4
GAI	15	27.3	14	25.5	14	25.5	12	21.8
CPI	19	34.5	12	21.8	17	30.9	7	12.7

Legend: FSIQ = Full-Scale IQ; VCI = Verbal Comprehension Index; PRI = Perceptual Reasoning Index; WMI = Working Memory Index; PSI = Processing Speed Index; GAI = General Ability Index; CPI = Cognitive Proficiency Index (CPI). Note: ^a^ *N* = 53; ^b^ *N* = 55.

**Table 6:** Bonferroni-corrected post-hoc tests comparing the WISC-IV primary indices.

**(a) Mother’s Sample**							
**Comparison**			**Mean Difference**	**SE**	**df**	**t**	**p_bonferroni_**
VCI	-	PRI	1.63	1.55	48	1.05	1
VCI	-	WMI	11.39	1.78	48	6.41	<.001
VCI	-	PSI	.20	2.42	48	.08	1
PRI	-	WMI	9.76	1.51	48	6.46	<.001
PRI	-	PSI	−1.43	2.09	48	−.68	1
WMI	-	PSI	−11.18	2.29	48	−4.88	<.001
**(b) Father’s Sample**							
**Comparison**			**Mean Difference**	**SE**	**df**	**t**	**p_bonferroni_**
VCI	-	PRI	−1.51	1.91	48	−.79	1
VCI	-	WMI	6.84	1.77	48	3.87	.009
VCI	-	PSI	1.86	1.67	48	1.11	1
PRI	-	WMI	8.35	1.92	48	4.34	.002
PRI	-	PSI	3.37	1.75	48	1.93	1
WMI	-	PSI	−4.98	1.95	48	−2.56	.385

Legend: VCI = Verbal Comprehension Index; PRI = Perceptual Reasoning Index; WMI = Working Memory Index; PSI = Processing Speed Index; GAI = General Ability Index; CPI = Cognitive Proficiency Index. Note: *N* = 49 (listwise)

**Table 7:** Pairwise correlations between parent WAIS-IV scores and child WISC-IV scores.

	(a) Mother-Child Correlations ^1^				(b) Father-Child Correlations ^2^			
	*r*	*p*	*ICC*	*CI 90%*	*r*	*p*	*ICC*	*CI 90%*
FSIQ	.26	(.029)	.17	[.01; .32]	−.01	(.514)	.00	[−.09;.10]
VCI	.16	(.124)	.10	[−.02; .23]	.08	(.271)	.04	[−.05; .14]
PRI	.25	(.034)	.14	[−.01; .28]	.15	(.132)	.08	[−.03; .19]
WMI	.22	(.058)	.22	[.07; .37]	.21	(.061)	.19	[.03; .34]
PSI	.31	(.013)	.25	[.10; .40]	−.05	(.644)	.00	[−.17; .17]
GAI	.24	(.045)	.10	[−.02; .23]	−.01	(.518)	.00	[−.06; .08]
CPI	.22	(.056)	.23	[.07; .38]	.01	(.460)	.01	[−.15; .18]

Legend: FSIQ = Full-scale IQ; VCI = Verbal Comprehension Index; PRI = Perceptual Reasoning Index; WMI = Working Memory Index; PSI = Processing Speed Index; GAI = General Ability Index; CPI = Cognitive Proficiency Index. ICC = Intraclass correlations. CI 90% = 90% confidence interval of the intraclass correlations. r = Pearson correlation. p = one-tailed significance level of Pearson correlation. Note: ^1^ *N* = 53 dyads. ^2^ *N* = 55 dyads.

**Table 8:** Descriptive statistics and tests of significant differences between WAIS-IV and WISC-IV scores in mother-child dyads.

	Children		Mothers		Tests of Significant Differences		
	*M*	*SD*	*M*	*SD*	*Diff.*	*t(52)*	*p*
FSIQ	132.36	7.74	123.38	9.49	8.98	6.19	<.001
VCI	131.66	8.86	120.81	9.64	10.85	6.59	<.001
PRI	133.00	10.72	119.32	10.46	13.68	7.70	<.001
WMI	114.15	9.82	109.68	10.65	4.47	2.54	.014
PSI	111.83	14.00	121.09	13.83	−9.26	−4.11	<.001
GAI	136.38	8.37	122.55	9.57	13.83	9.04	<.001
CPI	116.25	11.96	118.19	10.72	−1.94	−1.00	.323

Legend: VCI = Verbal Comprehension Index; PRI = Perceptual Reasoning Index; WMI = Working Memory Index; PSI = Processing Speed Index; GAI = General Ability Index; CPI = Cognitive Proficiency Index. Note: *N* = 53.

**Table 9:** Descriptive statistics and tests of significant differences between WAIS-IV and WISC-IV scores in father-child dyads.

	Children		Fathers		Tests of Significant Differences		
	*M*	*SD*	*M*	*SD*	*Diff.*	*t(52)*	*p*
FSIQ	133.38	8.22	119.42	13.12	13.96	6.67	<.001
VCI	132.07	9.11	116.00	13.29	16.07	7.71	<.001
PRI	134.15	10.99	118.73	11.53	15.42	7.80	<.001
WMI	114.62	10.80	110.64	15.15	3.98	1.77	.082
PSI	112.95	14.09	114.31	12.25	−1.36	−.53	.599
GAI	137.24	8.46	119.55	11.82	17.69	9.00	<.001
CPI	117.22	12.60	114.69	14.23	2.53	.99	.325

Legend: VCI = Verbal Comprehension Index; PRI = Perceptual Reasoning Index; WMI = Working Memory Index; PSI = Processing Speed Index; GAI = General Ability Index; CPI = Cognitive Proficiency Index. Note: *N* = 55.

## Data Availability

The data presented in this study are openly available from 30 September 2022, from OSF at https://osf.io/th3sq.
